# Altered spontaneous activity in young chronic cigarette smokers revealed by regional homogeneity

**DOI:** 10.1186/1744-9081-8-44

**Published:** 2012-08-22

**Authors:** Jinsong Tang, Yanhui Liao, Qijian Deng, Tieqiao Liu, Xiaogang Chen, Xuyi Wang, Xiaojun Xiang, Hongxian Chen, Wei Hao

**Affiliations:** 1 The Institute of Mental Health, the Second Xiangya Hospital, Central South University, 139 Renmin Road, Changsha, Hunan 410011, People’s Republic of China

**Keywords:** Chronic cigarette smokers, Regional homogeneity, Resting-state functional magnetic resonance imaging

## Abstract

**Background:**

Few studies have been previously published about the resting state brain activity in young chronic smokers, although many previous fMRI studies have shown that the task-related activity pattern is altered in chronic smokers.

**Methods:**

In the present study, forty-five healthy smokers (age: 27.9 ± 5.6 year) and forty-four healthy non-smoking control subjects (age: 26.3 ± 5.8 year) have been imaged with functional magnetic resonance imaging (fMRI) and analyzed with the regional homogeneity (ReHo) approach.

**Results:**

Compared with healthy controls, decreased ReHo was found in smokers in the right inferior frontal cortex and increased ReHo was found in the left superior parietal lobe (P < 0.01, 35 Voxels,Alphasim corrected).

**Conclusions:**

Our data suggested that, during resting state, neural function is less synchronized in the right inferior frontal cortex and more synchronized in the left superior parietal lobe in chronic smokers compared to non-smokers. The decreased synchronization in the right inferior frontal cortex may reflect lacking of control over reward-related behavior, and the increased synchronization may reflect smoking urges.

## Background

China, accounting for 30% of the world's smokers, is the nation with the largest population of smokers [1]. Chronic smoking in China causes a million deaths a year [2]. Total worldwide smoking related deaths are projected to rise from 5.4 million in 2005 to 6.4 million in 2030 [3].

Many functional magnetic resonance imaging (fMRI) studies have been performed to examine the effects of acute nicotine administration in smokers and non-smokers. A common finding from acute administration of nicotine/smoking is the globally reduced brain activity [4]. However, only a few studies [5-7] reported alteration of brain function activity during resting state in chronic smokers. The resting-state fMRI is a fundamental method to understand global and regional brain functional activity [8]. It has more potential applications in clinical studies than task-related fMRI which involves relatively complicated tasks.

Regional homogeneity (ReHo)[9] provides an approach to investigate local brain functional connectivity. The ReHo method measures local correlations in blood-oxygen-level-dependent (BOLD) time series, using Kendall’s coefficient of concordance (KCC) (Kendall and Gibbons., 1990). KCC is based on time course correlations between a given voxel and its neighbors. Thus, it can be used to measure the correlations between a number of time series of a given voxel and its nearest neighbors in a voxel-wise way. Findings from a recent study with 16 male heavy smokers suggested decreased synchronization in the prefrontal regions, as well as increased synchronization in the insula and the posterior cingulate cortex [7]. The aim of the present study was to further investigate the ReHo of spontaneous brain dynamics in young chronic smokers with a relatively large sample size.

## Materials and methods

### Subjects

The study sample comprised 45 smokers and 44 age-matched healthy volunteers (See Table 1 for participant demographics and smoking history). All these subjects were recruited from the local community by advertisements. They were initially screened during a semi-structured telephone interview to assess smoking, medical, psychiatric, medication, and substance use history. Smokers who had smoked 10 or more cigarettes per day during the previous year and had no period of smoking abstinence longer than 3 months in the past year, and met DSM-IV criteria for nicotine dependence were eligible for the study. All non-smokers had never smoked a cigarette before the study. More detailed information of subject recruitment procedures have been presented in a previous study [10]. 

**Table 1:** Demographic characteristics of the smokers and non-smokers

	**Smokers**	**non-smokers**
Demographic variables		
N	45	44
Age, years, mean ± SD	27.9 (5.6)	26.3 (5.8)
Range, years	19-39	19-38
Sex (female/male)	8/37 (17.8%)	10/34 (22.7%)
Subjects’ education, years, mean ± SD	13.1 ± 3.0 ^a^	15.0 ± 2.6
Handedness, right/left (n)	43/2	43/1
Married	19 (42.2%)	15 (34.1%)
Drinker/never-drinker ^b^	31/14	18/26
Age at start of smoking, mean ± SD	18.0 ± 4.3	―
Smoking initiation age range (years)	11-30	―
Years smoking, mean ± SD	10.2 ± 5.8	―
Range, years	1.5-21	―
Cigarettes per day	20.3 ± 7.6	
Range, cigarettes per day	10-40	―
Smoking cravings ^c^	6.41 ± 1.7	―
Range, scores	3-10	―

^a^ significantly different from control group, p < 0.01.

^b^ three participants reported drinking more than once a week among smokers and no non-smoking participants reported drinking more than once a week.

^c^ Before MRI acquisition run, participants were asked to rank their craving from 0 (“not at all”) to 10 (“extreme”).

Participants were excluded if they were a minority other than Han Chinese or had a diagnosis of mental retardation, current or past alcohol or drug abuse/dependence, a current or past central nervous system disease or condition, a medical condition or disease with likely significant central nervous system effects, history of head injury with skull fracture or loss of consciousness greater than 10 minutes, a physical problem that would render study measures difficult or impossible, any current or previous psychiatric disorder, a family history of a psychotic disorder, current or previous use of electroconvulsive therapy or psychotropic medications, or a positive pregnancy test. A licensed psychiatrist conducted all clinical interviews. The Second Xiangya Hospital of Central South University Review Board approved all procedures used. The studies were carried out in accordance with the Declaration of Helsinki. Subjects were fully informed about the measurement and MRI scanning in the study. Written informed consent was given by all study participants.

### MRI Data acquisition

Resting state functional magnetic resonance (MR) images were acquired using a 3.0-Tesla Siemens scanner (Allegra; Siemens Medical System) at the Magnetic Resonance Center of Hunan Provincial People's Hospital. Foam pads were used to reduce head movements and scanner noise. Participants were required simply to keep still as much as possible, close their eyes and not to think of anything systematically. The resting-state functional images were acquired by using an echo-planar imaging sequence with the following parameters: 36 axial slices, thickness/skip = 3/1 mm, in-plane resolution =64 × 64, repetition time = 3000 ms, echo time = 30 ms, flip angle = 90, field of view = 220 × 220 mm, 180 volumes.

### Data analysis

The first 10 time points of the fMRI data were discarded because of the instability of the initial MRI signal and the adaptation of the subjects to the scanning environment. The remaining 170 images were pre-processed using Statistical Parametric Mapping 5 (SPM5; http://www.fil.ion.ucl.ac.uk/spm/). They were slice-time-corrected, and aligned to the first image of each session for motion correction, spatially normalized to the Montreal Neurological Institute (MNI) EPI template in SPM5, and each voxel was resampled to 3 × 3 × 3 mm^3^. Datasets with more than 1.5 mm maximum translation in x, y, or z, or 1 degree of maximum rotation about three axes were discarded. Linear detrending and temporal bandpass filtering (0.01–0.08 Hz) were carried out using REST software[11] ( http://resting-fmri.sourceforge.net ).

Individual ReHo maps were generated by calculating Kendall's coefficient of concordance (KCC, also called ReHo value) for the time series of a given voxel and those of its nearest neighbors (26 voxels), on a voxel-wise basis. The intracranial voxels were extracted to make a mask. For standardization purposes, each individual ReHo map was divided by its own mean ReHo within the mask. Then, the data were smoothed with a Gaussian filter of 6 mm full width at half-maximum (FWHM) to reduce noise and residual differences in gyral anatomy. All these analyses were carried out using REST software.

To explore ReHo differences between the groups, a second-level random-effect two-sample t-test was performed on individual normalized ReHo maps in a voxel-by-voxel manner. Age, gender and education level were entered as covariates of no interest. Correction for multiple comparisons was performed using Monte Carlo simulation. A corrected threshold of p < 0.01 (two-tailed) was derived from a combined threshold of p < 0.01 for each voxel and a cluster size of > 35 voxels was determined using the AlphaSim program in AFNI software (Parameters: single voxel p < 0.01, 5000 simulations, FWHM = 6 mm, with gray matter mask, http://afni.nimh.nih.gov/). Subsequently, we performed a post-hoc correlation analysis in order to investigate the relationship between ReHo values of the significant clusters and clinical factors. Correlations between ReHo values of the significant clusters and clinical factors including age of starting smoking, duration (months) of smoking, accumulative smoked cigarettes, quantity of cigarette use per day and craving for cigarettes,were calculated by partial correlation analysis controlling for age, gender and education level (two tail, p <0.05).

## Results

The results were present for 45 smokers and 44 non-smokers. The overall sample was characterized typically by middle-upper class socioeconomic status in China. Groups were well matched in age, gender and handedness though there was a difference in educational levels (p < 0.01). Basic characteristics of subjects were detailed in Table 1.

**Figure 1:**
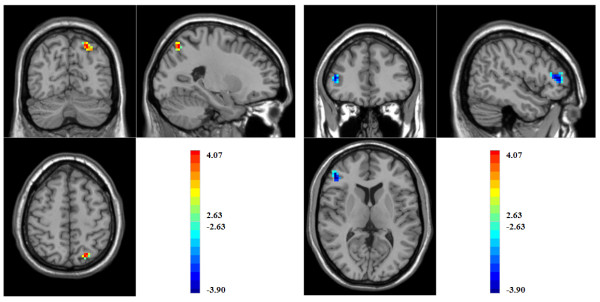
**Brain areas of ReHo difference between smokers and non-smokers.** Warm and cool colors indicate smokers-related ReHo increase in left side of the brain and decrease in right side of the brain, respectively. (P < 0.01, 35 Voxels,Alphasim corrected).

In comparison with non-smokers, chronic smokers displayed significantly decreased ReHo in the right inferior frontal cortex and increased ReHo in the left superior parietal lobe (as shown in Table 2. and Figure 1.). In post hoc analysis, there were no significant correlations between mean regional ReHo values and clinical characteristics (all p’s > 0.1).

**Table 2:** Brain areas of ReHo changes in smokers in comparison with non-smokers (P < 0.01, 35 Volexs,Alphasim corrected)

**Brodmann area**	**Brain region**	**MNI coordinates**			**T**	**Cluster size**
		**X**	**Y**	**Z**		
46	ReHo decreased in right Inferior frontal gyrus	48	39	9	3.88	57
7	ReHo increased in left Superior parietal gyrus	−24	−69	54	4.07	53

## Discussion

In this study, we found decreased ReHo in the right inferior frontal cortex and increased ReHo in left superior parietal lobe in chronic smokers during resting state, which suggests that, during resting state, neural function is less synchronized in the right inferior frontal cortex and more synchronized in the left superior parietal lobe in chronic smokers compared to non-smokers.

This study observed decreased ReHo for chronic smokers in the right inferior frontal cortex. The frontal cortex is involved in compulsive drug-seeking behaviors in drug dependence [12]. Neuroimaging studies indicated that the frontal cortex has been affected directly by long-term exposure to addictive drugs [13]. This hypo-function of the frontal region [14] (e.g. lack of control over reward-related behavior) also existed in nicotine dependent individuals. The right inferior frontal gyrus has been implicated in response inhibition [15]. Recently, Yu et al. investigated the ReHo values in male heavy smokers for the first time [7]. In Yu’s study, they found heavy smokers exhibited decreased ReHo in right inferior frontal gyrus, and increased ReHo in the insula and the posterior cingulate cortex [7]. In our study, we also found decreased ReHo in the right inferior frontal cortex, which is similar to results seen in Yu’s study. However, results are inconsistent in other brain regions. Demographic characteristics of the smokers in our study and Yu’s study, such as the mean age (27.9 VS 41.6) and the mean years of smoking (10.2 VS 21.1), may partly explain the inconsistent results. In addition, a large body of evidence has shown that the prefrontal cortex plays a critical role in cognitive function [16,17] and that chronic cigarette smoking is associated with neurocognitive (such as executive skills, learning and memory, processing speed, and working memory) deficits [18,19]. Therefore, it was not unexpected that ReHo decreased in the inferior frontal cortex in chronic smoking individuals.

The Parietal lobe is a crucial region of planning and executing tool use movements [20]. In a recent Meta-analysis, the parietal lobe was found to be related to the reward-circuit [21], which suggests that the parietal cortex can be involved in addiction behavior. A previous task-related fMRI study have found abnormal parietal cortex activations in amphetamine dependent individuals [22]. In the current study, we detected increased ReHo in the left superior parietal lobe in smokers compared with non-smokers. ReHo provided information about the connectivity at the local level [23]. Increased ReHo may reflect increased local synchronization in neighboring voxels might be associated with high regional metabolism. In the present study, the enhanced synchronization in local regional resting-state blood oxygen level dependent BOLD activity detected in chronic smokers in the parietal cortex may reflect atypical smoking urges.

Some limitations are worth mentioning. Education levels were not well matched in the two groups. However, education level was set as a covariate of no interest in the group analysis. In addition, we didn’t evaluate sex effects on outcome measures since only a few female subjects (n = 8) were in this study.

In summary, the present study found decreased ReHo in the right inferior frontal cortex and increased ReHo in the left superior parietal lobe in the chronic smoking group compared to the non-smoking group during the resting state. These results may better our understanding of the neurobiological consequences of chronic smoking.

## Competing interests

The authors declare that they have no competing interests.

## Authors’ contributions

Design: JT, YL, WH Data collection: JT, YL, QD, TL, XC, XW, XX, HC Analysis: JT, YL Writing: JT, YL All authors read and approved the final manuscript.
